# Assessing Differences Between Results Determined According to the Guide to the Expression of Uncertainty in Measurement

**DOI:** 10.6028/jres.115.031

**Published:** 2010-12-01

**Authors:** Raghu N. Kacker, Rüdiger Kessel, Klaus-Dieter Sommer

**Affiliations:** National Institute of Standards and Technology, Gaithersburg, MD 20899, USA; Physikalisch-Technische Bundesanstalt, D-38116 Braunschweig, Germany

**Keywords:** Birge test, interlaboratory evaluations, predictive *p*-value, uncertainty

## Abstract

In some metrology applications multiple results of measurement for a common measurand are obtained and it is necessary to determine whether the results agree with each other. A result of measurement based on the Guide to the Expression of Uncertainty in Measurement (GUM) consists of a measured value together with its associated standard uncertainty. In the GUM, the measured value is regarded as the expected value and the standard uncertainty is regarded as the standard deviation, both known values, of a state-of-knowledge probability distribution. A state-of-knowledge distribution represented by a result need not be completely known. Then how can one assess the differences between the results based on the GUM? Metrologists have for many years used the Birge chisquare test as ‘a rule of thumb’ to assess the differences between two or more measured values for the same measurand by pretending that the standard uncertainties were the standard deviations of the presumed sampling probability distributions from random variation of the measured values. We point out that this is misuse of the standard uncertainties; the Birge test and the concept of statistical consistency motivated by it do not apply to the results of measurement based on the GUM. In 2008, the International Vocabulary of Metrology, third edition (VIM3) introduced the concept of metrological compatibility. We propose that the concept of metrological compatibility be used to assess the differences between results based on the GUM for the same measurand. A test of the metrological compatibility of two results of measurement does not conflict with a pairwise Birge test of the statistical consistency of the corresponding measured values.

## 1. Introduction

To test the proficiency of individual laboratories in conducting specific tasks, interlaboratory comparisons (ILC) are often used. In ILC between measurement laboratories, the task is generally the measurement of a common artifact or fractions of the same sample of material. To develop a certified reference material, a well characterized material is measured by two or more methods in one or more laboratories. In both cases the data consist of multiple results of measurement (measured values with associated uncertainties) of a common measurand. To assess the differences between two or more measured values for the same measurand, metrologists have for many years used a test proposed by physicist Raymond T. Birge in 1932 [1]. Birge introduced the term consistency for lack of significant differences between measured values. The Birge test is based on treating the measured values as realizations of random draws from sampling probability density functions (pdfs). A sampling pdf models possible outcomes for measured values in contemplated replications of the measurement procedure in the same conditions. Therefore, the consistency of measured values assessed by the Birge test is statistical consistency. The Birge test applies to uncorrelated measured values only. In Sec. 2, we review a concept of statistical consistency motivated by the Birge test. The idea of statistical consistency belongs to the period when the error analysis view of measurements was prevalent. The error analysis view of measurements was a hindrance to communicating the results of measurement and in advancing the science and technology of measurement. Therefore leading authorities in the field of metrology developed the Guide to the Expression of Uncertainty in Measurement (GUM) [2]. According to the GUM, a result of measurement consists of a measured value together with its associated standard uncertainty. In the GUM, the measured value is regarded as the expected value and the standard uncertainty is regarded as the standard deviation, both known values, of a state-of-knowledge probability distribution. A state-of-knowledge distribution represented by a result of measurement need not be completely known. We note in Sec. 3 that the Birge test and the concept of statistical consistency motivated by it are not applicable to the results of measurement based on the GUM. Then how can one assess the differences between results based on the GUM for the same measurand? In 2008, the International Vocabulary of Metrology, third edition (VIM3) [3] introduced the concept of metrological compatibility of two or more results of measurement determined according to the (GUM). In Sec. 4, we review the VIM3 concept of metrological compatibility and propose that this concept be used to assess the differences between multiple results based on the GUM for the same measurand. In Sec. 5, we show that a test of the metrological compatibility of two results of measurement does not conflict with a pairwise Birge test of the statistical consistency of the corresponding measured values.

## 2. The Birge Test and Concept of Statistical Consistency

Suppose *x*_1_, …, *x_n_* are *n* measured values for a common measurand which is believed to be sufficiently stable. The Birge test is based on regarding the measured values *x*_1_, …, *x_n_* as realizations of random draws from their presumed sampling pdfs. A sampling pdf models possible outcomes in contemplated replications of a measurement procedure subject to random effects in the same conditions. Therefore, the consistency (lack of significant differences between measured values) assessed by the Birge test is statistical consistency. The Birge test is applicable when the sampling pdfs of the measured values *x*_1_, …, *x_n_* are uncorrelated. The Birge test requires knowledge of the variances *σ*_1_^2^, …, *σ_n_*^2^ of the sampling pdfs of respectively. Statistical consistency of the measured values *x*_1_, …, *x_n_* means that their expected values are indistinguishable^1^ in view of the corresponding variances. Specifically, the Birge test checks whether the measured values *x*_1_, …, *x_n_* may be modeled as realizations from normal (Gaussian) sampling pdfs with unknown but equal expected values and known variances *σ*_1_^2^, …, *σ_n_*^2^. Birge proposed that to check the consistency of the measured values *x*_1_, …, *x_n_*, one can calculate the test statistic

(1) $$R^{2}=\sum_{i=1}^{n}w_{i}{\left(x_{i}-x_{w}\right)}^{2}/(n-1),$$

where *w_i_* = 1/*σ_i_*^2^, for *i* = 1, 2, …, *n*, and *x*_W_ = Σ*_i_ w_i_ x_i_*/Σ*_i_ w_i_* is the weighted mean of *x*_1_, …, *x_n_*. If the calculated value of *R*^2^ is substantially larger than one, then the dispersion of *x*_1_, …, *x_n_* is greater than what can be expected from the normal pdfs with equal expected values and known variances *σ*_1_^2^, …, *σ_n_*^2^. In that case the measured values *x*_1_, …, *x_n_* can be declared to be statistically inconsistent.

### Statistical interpretation of the Birge test

Birge was a physicist and he proposed his test independently of and before much of the statistical theory as it is known today was established. However, the Birge test of consistency can now be interpreted as a classical (sampling theory) statistical test of hypothesis. The measured values *x*_1_, …, *x_n_* are presumed to have normal sampling pdfs with unknown but equal expected values and variance-covariance matrix *τ*^2^ × Diag [*σ*_1_^2^, …, *σ_n_*^2^], where *τ*^2^ is an unknown parameter and *σ*_1_^2^, …, *σ_n_*^2^ are known. The null hypothesis H_0_ is that *τ*^2^ ≤ 1 and the alternative hypothesis H_1_ is that *τ*^2^ > 1. The null hypothesis H_0_ means that the variances of *x*_1_, …, *x_n_* are not greater than *σ*_1_^2^, …, *σ_n_*^2^, respectively. The alternative hypothesis H_1_ means that the variances of *x*_1_, …, *x_n_* are greater than *σ*_1_^2^, …, *σ_n_*^2^ [4]. The classical *p*-value *p*_C_ is the maximum probability under the null hypothesis of realizing in contemplated replications of the *n* measurements a value of the test statistic more extreme than its realized (calculated) value. The classical *p*-value of a realization of (*n* – 1) *R*^2^ is

(2) $$p_{C}=\mathrm{Pr}\{\chi_{\left(n-1\right)}^{2}\geq (n-1)R^{2}\},$$

where *χ*^2^_(_*_n_*_–1)_ denotes a variable with the chi-square probability distribution with degrees of freedom (*n* – 1) [4]. If the classical *p*-value *p*_C_ is too small, say, less than 0.05, then the null hypothesis is rejected with level of significance 0.05 or less. A rejection of the null hypothesis means that the dispersion of the measured values *x*_1_, …, *x_n_* is greater than what can be expected from normal distributions for *x*_1_, …, *x_n_* with equal expected values and stated variances *σ*_1_^2^, …, *σ_n_*^2^, respectively. The dispersion of *x*_1_, …, *x_n_* can be greater than expected under the null hypothesis because either the variances of *x*_1_, …, *x_n_* are greater than *σ*_1_^2^, …, *σ_n_*^2^ or their expected values are not equal. If the stated variances *σ*_1_^2^, …, *σ_n_*^2^ are not questionable then the assumption that the expected values of *x*_1_, …, *x_n_* are equal appears to be unreasonable. In that case, the measured values *x*_1_, …, *x_n_* can be declared to be statistically inconsistent.

### Limitations of the Birge test

A limitation of the Birge test is that it is applicable for uncorrelated measured values *x*_1_, …, *x_n_* only. However, it can be easily generalized to correlated measured values *x*_1_, …, *x_n_* whose covariances denoted by *σ*_1_^2^, …, *σ*_(_*_n_*_–1)_*_n_* are known [4]. The Birge test suggests the following notion of the statistical consistency of the measured values *x*_1_, …, *x_n_*: The measured values ***x*** = (*x*_1_, …, *x_n_*)^t^ are said to be statistically consistent if their dispersion *is not greater than* what can be expected from the *normal consistency model* which postulates that the joint *n*-variate sampling pdf of ***x*** is normal N(**1***μ*, ***D***) with unknown expected value **1***μ* and variance-covariance matrix ***D*** = [*σ_ij_*], where **1** = (1, …, 1)^t^, *σ_ij_* is the covariance between *x_i_* and *x_j_*, and *σ_ii_* = *σ_i_*^2^ for *i*, *j* = 1, 2, …, *n* [4].

Another limitation of the Birge test (and of its generalized version for correlated measured values) is that it is a one sided test of hypothesis which checks whether the dispersion of *x*_1_, …, *x_n_* is more than what can be expected from a normal consistency model. A review of the Birge test in [5] notes that if the realized value of the Birge test statistic *R*^2^ is substantially less than one, then the stated variances *σ*_1_^2^, …, *σ_n_*^2^ may well be too large. To avoid declarations of statistical consistency from overstated variances, the following definition of statistical consistency was proposed in [6].

### Definition of statistical consistency

The measured values ***x*** = (*x*_1_, …, *x_n_*)^t^ are said to be statistically consistent if they *reasonably fit* the normal consistency model which postulates that the joint *n*-variate sampling pdf of ***x*** is normal N(**1***μ*, ***D***) with unknown expected value **1***μ* and variance-covariance matrix ***D*** = [*σ_ij_*].

This definition requires a different approach for testing statistical consistency than the Birge test and its generalized version for correlated values. A modern method to assess the fit of a statistical model to the data is Bayesian posterior predictive checking [6]. Posterior predictive checking is a Bayesian adaptation of the classical (sampling theory) statistical hypothesis testing. A function of the data (and possibly unknown parameters) called ‘discrepancy measure’ is defined to characterize a potential discrepancy between the statistical model and the data. The posterior predictive *p*-value *p*_P_ of adiscrepancy measure T(***x***) is the probability of realizing in contemplated replications a value of the discrepancy measure more extreme than its realized value. If the posterior predictive *p*-value is close to zero (or to one) then the fit of the statistical model to data is suspect.

If the measured values *x*_1_, …, *x_n_* were uncorrelated, then the statistic T_c_(***x***) = (*n* – 1) *R*^2^ = Σ*_i_ w_i_* (*x_i_* – *x*_W_)^2^ is a useful discrepancy measure to check the overall fit of the normal consistency model N(**1***μ*, ***D***) to the measured values *x*_1_, …, *x_n_*. As discussed in [6, Sec. 2.4], the posterior predictive *p*-value of the realized discrepancy measure T_c_(***x***) = (*n* – 1) *R*^2^ is

(3) $$p_{P}=\mathrm{Pr}\{\chi_{\left(n-1\right)}^{2}\geq (n-1)R^{2}\}.$$

We note that (3) is identical to the classical *p*-value *p*_C_ given in (2). Thus Bayesian posterior predictive checking of the discrepancy measure T_c_(***x***) = (*n* – 1) *R*^2^ is equivalent to the Birge test of statistical consistency.

Bayesian posterior predictive checking can be used to investigate any number of potential discrepancies between the statistical model and the data. To assess the difference between two particular measured values *x_i_* and *x_j_*, the statistic T*_i – j_*(***x***) = | *x_i_* – *x_j_*| is a useful discrepancy measure, for *i*, *j* = 1, 2, …, *n* and *i* ≠ *j*. The Bayesian posterior predictive *p*-value of the realized discrepancy measure |*x_i_*–*x_j_*| is

(4) $$p_{P}=\mathrm{Pr}\{Z\geq \frac{\left|x_{i}-x_{j}\right|}{\sqrt{\sigma_{i}{}^{2}+\sigma_{j}{}^{2}-2\rho_{ij}\sigma_{i}\sigma_{j}}}\},$$

where *ρ_ij_* is the correlation coefficient between the presumed normal sampling pdfs of *x_i_* and *x_j_*; the covariance between *x_i_* and *x_j_* is *σ_ij_* = *ρ_ij_ σ_i_ σ_j_*, and *Z* denotes a variable with standard normal distribution N(0, 1) [6, Sec. 3.2]. A posterior predictive *p*-value *p*_P_ close to zero suggests that the difference between *x_i_* and *x_j_* is larger than what can be expected from the normal statistical consistency model N(**1***μ*, ***D***). That is, the measured values *x_i_* and *x_j_* do not seem to have the same expected value and hence they are not mutually statistically consistent.

## 3. Concept of Statistical Consistency Does Not Apply to Results Based on the GUM

A result of measurement determined according to the GUM consists of a measured value together with its associated standard uncertainty. Suppose [*x*_1_, *u*(*x*_1_)], …, [*x_n_*, *u*(*x_n_*)] are *n* results of measurement for a common measurand, where *x*_1_, …, *x_n_* are the measured values and *u*(*x*_1_), …, *u*(*x_n_*) are the corresponding standard uncertainties. According to the GUM, a measured value *x_i_* and its associated standard uncertainty *u*(*x_i_*) represent a state-of-knowledge pdf attributed to the measurand, for *i* = 1, 2, …, *n*. Following the GUM, we use the symbol *X_i_* for a quantity as well as for a variable with a state-of-knowledge pdf about the quantity *X_i_* represented by the result [*x_i_*, *u*(*x_i_*)], for *i* = 1, 2, …, *n*. The measured value *x_i_* is regarded as the expected value E(*X_i_*) and the standard uncertainty *u*(*x_i_*) is regarded as the standard deviation S(*X_i_*) of the pdf of *X_i_*, for *i* = 1, 2, …, *n*. The mainstream GUM requires knowledge of only the expected value E(*X_i_*) and the standard deviation S(*X_i_*) of a state-of-knowledge pdf of *X_i_*. The GUM does not require that the state-of-knowledge pdf of *X_i_* be completely known. When the state-of-knowledge pdfs of *X*_1_, …, *X_n_* are correlated, the correlation coefficients are assumed to be known. Following the GUM we denote the correlation coefficient R(*X_i_*, *X_j_*) between the state-of-knowledge pdfs of *X_i_* and *X_j_* by the symbol *r*(*x_i_*, *x_j_*). Note that {*x*_1_, …, *x_n_*}, {*u*(*x*_1_), …, *u*(*x_n_*)}, and {*r*(*x*_1_, *x*_2_), …, *r*(*x*_(_*_n_*_–1)_, *x_n_*)} are symbols for known values.

For many years, metrologists have used the Birge test as ‘a rule of thumb’ to assess the consistency of the measured values by treating the squared standard uncertainties *u*^2^(*x*_1_), …, *u*^2^(*x_n_*) as the known variances *σ*_1_^2^, …, *σ_n_*^2^ of the presumed normal (Gaussian) sampling pdfs of the measured values *x*_1_, …, *x_n_*; see, for example [8]. The guideline for the analysis of key comparisons developed by the BIPM Director’s Advisory Group on Uncertainties recommends the use of Birge chi-square test to assess the consistency of measured values by treating the squared standard uncertainties as the known variances of the presumed sampling pdfs of the measured values [9]. The consistency of the measured values from CIPM key comparisons and supplementary comparisons is almost always assessed using the Birge test [10].

The squared standard uncertainties *u*^2^(*x*_1_), …, *u*^2^(*x_n_*) cannot in any logical sense be identified with the known variances *σ*_1_^2^, …, *σ_n_*^2^ of the presumed normal (Gaussian) sampling pdfs of the measured values *x*_1_, …, *x_n_*. The standard deviation of a sampling pdf represents possible dispersion from random variation in contemplated replications of the measurement procedures. A standard uncertainty expresses the dispersion of a state-of-knowledge pdf which could be attributed to the measurand based on all available statistical and non-statistical information. A standard uncertainty includes all significant components whether arising from random effects or from corrections applied for systematic effects. All available statistical and non-statistical information is used to evaluate a standard uncertainty. In measurements done in high echelon laboratories, the component of uncertainty arising from random effects is generally a very small part of the combined standard uncertainty. Treating the squared standard uncertainties *u*^2^(*x*_1_), …, *u*^2^(*x_n_*) determined according to the GUM as the known variances *σ*_1_^2^, …, *σ_n_*^2^ from random variation (in contemplated replications of the measurements) is a misuse of the standard uncertainties. Also, as noted earlier, the state-of-knowledge pdfs represented by the results [*x*_1_, *u*(*x*_1_)], …, [*x_n_*, *u*(*x_n_*)] may not be completely known. Therefore the Birge test and the concept of statistical consistency motivated by the Birge test do not apply to the results of measurement determined according to the GUM.

## 4. VIM3 Concept of Metrological Compatibility Applies to Results Based on the GUM

A measured quantity value [3, definitions 1.19 and 2.10] is a product of a numerical value and a measurement unit. The measurement unit implies that the measured value is traceable to a reference for that measurement unit. A result of measurement (measured value together with its associated standard uncertainty) is traceable to a reference only if the result can be related to a practical realization of that reference through a documented unbroken chain of calibrations each contributing to the measurement uncertainty [3, definition 2.41]. Two or more results of measurement are *metrologically comparable* only if they are traceable to the same reference [3, definition 2.46]. Metrological comparability does not imply that the measured values have similar magnitudes. Thus, for example, distance between my apartment and my office expressed in meters is metrologically comparable to the distance between my apartment and the moon also expressed in meters. The concept of metrological compatibility discussed in the next section applies only to those results of measurement for a common measurand which are metrologically comparable. That is, the results must be traceable to the same reference.

The concept of statistical consistency can be applied to any set of numerical values which have similar magnitudes. They do not have to be measured values. Thus, for example, one can test statistical consistency of deviations (or relative deviations expressed as percentage) from a benchmark value. Although a metrologist is expected to assess consistency of only those measured values which have the same measurement unit, it is not a requirement of statistical consistency.

All *n* results [*x*_1_, *u*(*x*_1_)], …, [*x_n_*, *u*(x*_n_*)] for a common measurand must be traceable to the same reference for them to be metrologically comparable [3, definition 2.46]. The VIM3 concept of metrological compatibility is defined for two results of measurement at a time. The following definition is an elaboration of the succinct definition given in VIM3 [3, definition 2.47].

### Definition of metrological compatibility

Two metrologically comparable results [*x*_1_, *u*(*x*_1_)] and [*x*_2_, *u*(*x*_2_)] for the same measurand are said be metrologically compatible if

(5) $$\zeta (x_{1}-x_{2})=\frac{\left|x_{1}-x_{2}\right|}{\sqrt{u^{2}(x_{1})+u^{2}(x_{2})-2r(x_{1},x_{2})u(x_{1})u(x_{2})}}\leq \kappa ,$$

for a specified threshold *κ*, where *r*(*x*_1_, *x*_2_) is a symbol for the correlation coefficient R(*X*_1_, *X*_2_) between the variables *X*_1_ and *X*_2_. The quantity in the denominator of (5) is the standard deviation of the state-of-knowledge pdf for *X*_1_ – *X*_2_, which may be incompletely determined. When the pdfs represented by [*x*_1_, *u*(*x*_1_)] and [*x*_2_, *u*(*x*_2_)] are uncorrelated, then R(*X*_1_, *X*_2_) = 0 and (5) reduces to

(6) $$\zeta (x_{1}-x_{2})=\frac{\left|x_{1}-x_{2}\right|}{\sqrt{u^{2}(x_{1})+u^{2}(x_{2})}}\leq \kappa .$$

A set of metrologically comparable results [*x*_1_, *u*(*x*_1_)], [*x*_2_, *u*(*x*_2_)], …, [*x_n_*, *u*(*x_n_*)] for the same measurand is said be metrologically compatible if for every one of the *n*(*n* – 1)/2 pairs of results [*x_i_*, *u*(*x_i_*)] and [*x_j_*, *u*(*x_j_*)] we have

(7) $$\zeta (x_{i}-x_{j})=\frac{\left|x_{i}-x_{j}\right|}{\sqrt{u^{2}(x_{i})+u^{2}(x_{j})-2r(x_{i},x_{j})u(x_{i})u(x_{j})}}\leq \kappa ,$$

for a specified threshold *κ* [3, definition 2.47]. The VIM3 does not discuss how the threshold *κ* should be determined. A conventional value of *κ* is two.

The concept of metrological compatibility can be used to assess the differences between the results of measurement based on the GUM for the same measurand. The concepts of metrological comparability and compatibility do not require that the state-of-knowledge pdfs represented by the results [*x*_1_, *u*(x_1_)], [*x*_2_, *u*(*x*_2_)], …, [*x_n_*, *u*(*x_n_*)] be completely known. Thus they fit the GUM. When the set of results [*x*_1_, *u*(*x*_1_)], …, [*x_n_*, *u*(*x_n_*)] is metrologically compatible, we can say that the differences between the measured values *x*_1_, …, *x_n_* are insignificant in view of the uncertainties *u*(*x*_1_), …, *u*(*x_n_*).

To assess metrological compatibility of results based on the GUM using the criteria (5), (6), or (7)), the threshold *κ* needs to be specified. A proper choice of *κ* is to a large extent a matter of agreement because it requires accepting the economic consequences of that choice. Although a conventional value of *κ* is two, depending on the application, the interested parties could agree on a different value for *κ*. Once the value of the threshold *κ* is set the conclusion of a test of metrological compatibility based on the VIM3 definition is dichotomous, either a set of results is metrologically compatible or incompatible. The concept of metrological compatibility is being used by metrologists who are familiar with it; see for example [11, 12].

The VIM3 definition of metrological compatibility can be easily extended to metrological compatibility of a set of results and a reference result [*x*_R_, *u*(*x*_R_)], where *x*_R_ is the reference value with standard uncertainty *u*(*x*_R_). Suppose the pdfs represented by the measurement results are uncorrelated with the pdf represented by the reference result. A set of results [*x*_1_, *u*(*x*_1_)], …, [*x_n_*, *u*(*x_n_*)] metrologically comparable with a reference result [*x*_R_, *u*(*x*_R_)] is compatible if

(8) $$\zeta (x_{i}-x_{R})=\frac{\left|x_{i}-x_{R}\right|}{\sqrt{u^{2}(x_{i})+u^{2}(x_{R})}}\leq \kappa ,$$

for *i* = 1, 2, …, *n* [13]. Similarly a set of results [*x*_1_, *u*(*x*_1_)], …, [*x_n_*, *u*(*x_n_*)] metrologically comparable with a combined result [*x*_C_, *u*(*x*_C_)], where *x*_C_ is the combined value (such as arithmetic mean or a weighted mean) with standard uncertainty *u*(*x*_C_) is compatible if

(9) $$\zeta (x_{i}-x_{C})=\frac{\left|x_{i}-x_{C}\right|}{\sqrt{u^{2}(x_{i})+u^{2}(x_{C})-2r(x_{i},x_{C})u(x_{i})u(x_{C})}}\leq \kappa ,$$

where *r*(*x_i_*, *x*_C_) denotes the correlation coefficient between the pdfs represented by [*x_i_*, *u*(x*_i_*)] and [*x*_C_, *u*(*x*_C_)], for *i* = 1, 2, …, *n* [13].

## 5. Concluding Remarks

For many years, metrologists have used the Birge chi-square test as ‘a rule of thumb’ to assess the differences between two or more measured values for the same measurand by pretending that the squared standard uncertainties were the known variances of the presumed normal sampling pdfs of the measured values. This is misuse of the standard uncertainties based on the GUM. The Birge test and the concept of statistical consistency do not apply to the results of measurement based on the GUM. As discussed in this paper, the VIM3 concept of metrological compatibility can be used to assess the differences between the results of measurement determined according to the GUM. Thus metrologists can start using the VIM3 concept of metrological compatibility in place of the Birge test to assess the differences between multiple results of measurement of the same measurand.

The following is a pertinent question. Could the conclusions (about mutual agreement of results) based on the VIM3 concept of metrological compatibility and the Birge test (based on treating squared standard uncertainties as the known variances of sampling pdfs of measured values) differ? It is difficult to directly compare the Birge test and a test of metrological compatibility because the former is defined for an arbitrary positive integer *n* > 1 and the latter is defined for only two results at a time. For pairwise comparisons (*n* = 2), the Birge test statistic *R*^2^ = Σ*_i_ w_i_* (*x_i_* – *x*_W_)^2^/(*n* – 1) reduces to

(10) $$R^{2}=\frac{{\left(x_{1}-x_{2}\right)}^{2}}{\left(\sigma_{1}{}^{2}+\sigma_{2}{}^{2}\right)},$$

which is square of (*x*_1_ – *x*_2_)/√(*σ*_1_^2^ + *σ*_2_^2^). Under the null hypothesis that the presumed normal sampling pdfs of *x*_1_ and *x*_2_ have the same expected value, the distribution of (*x*_1_ – *x*_2_)/√(*σ*_1_^2^ + *σ*_2_^2^) is normal N(0, 1). Therefore when *n* = 2, the normal distribution can be used to assess the absolute difference | *x*_1_ – *x*_2_ |. The square of a normal N(0, 1) variable has the chi-square distribution *χ*^2^_(1)_ with degrees of freedom 1. Therefore the square of the (1 – *α*/2) × 100-th percentile z_[1–_*_α_*_/2]_ of normal N(0, 1) distribution is equal to the (1 – *α*) × 100-th percentile *χ*^2^_(1)[1–_*_α_*_]_ of *χ*^2^_(1)_ distribution. Thus the realized value of (10) being less than *χ*^2^_(1)[1–_*_α_*_]_ is equivalent to the ratio (*x*_1_ – *x*_2_)/√(*σ*_1_^2^ + *σ*_2_^2^) being less than z_[1–_*_α_*_/2]_. It follows that declaration of Birge statistical consistency when the classical *p*-value *p*_C_ of the Birge test (2) is less than 0.05 (for example) is equivalent to the realization that

(11) $$\frac{\left|x_{1}-x_{2}\right|}{\sqrt{\left(\sigma_{1}{}^{2}+\sigma_{2}{}^{2}\right)}}\leq z_{\left[0.975\right]}=1.96\approx 2.$$

We note from (6) and (11) that if the threshold *κ* for metrological compatibility is set as *κ* = 2 then the conclusion of a check of metrological compatibility between a pair of results [*x*_1_, *u*(*x*_1_)] and [*x*_2_, *u*(*x*_2_)] would be identical to the assessment of statistical consistency between *x*_1_ and *x*_2_ based on the Birge test by (wrongly) treating *u*^2^(*x*_1_) and *u*^2^(*x*_2_) as *σ*_1_^2^ and *σ*_2_^2^, respectively (and treating the correlation coefficient R(*X*_1_, *X*_2_) as *ρ*_12_ which is zero in the Birge test). Therefore a pairwise Birge test of statistical consistency and a test of metrological compatibility do not conflict.

## Footnotes

1 In statistical literature the term consistency is applied to a statistical estimator. A point statistical estimator is said to be consistent if it approaches the parameter being estimated as the sample size increases.
